# Interrogating the Genomic Landscape of Uterine Leiomyosarcoma: A Potential for Patient Benefit

**DOI:** 10.3390/cancers14061561

**Published:** 2022-03-18

**Authors:** Genevieve V. Dall, Anne Hamilton, Gayanie Ratnayake, Clare Scott, Holly Barker

**Affiliations:** 1 Walter and Eliza Hall, Institute of Medical Research, Parkville, VIC 3052, Australia; scottc@wehi.edu.au (C.S.); barker.h@wehi.edu.au (H.B.); 2 Department of Medical Biology, University of Melbourne, Parkville, VIC 3010, Australia; anne.hamilton@petermac.org; 3 Peter MacCallum Cancer Centre, Melbourne, VIC 3000, Australia; 4 Royal Women’s Hospital, Parkville, VIC 3052, Australia; gayanie.ratnayake@rch.org.au

**Keywords:** uterine leiomyosarcoma, sarcoma, rare cancer, gynaecological cancer, targeted therapy, clinical trials, preclinical models

## Abstract

**Simple Summary:**

Uterine leiomyosarcoma is an aggressive and rare cancer that is difficult to treat. There are a number of mutations that are common to uterine leiomyosarcoma that are currently not routinely targeted therapeutically in this cancer type. In this review, we summarise the studies being undertaken to investigate the effectiveness of targeting these mutations either pre-clinically in models of uterine leiomyosarcoma or in other cancers in the clinic. We hope this review will encourage the inclusion of uterine leiomyosarcoma in clinical trial design, which in turn will lead to improved survival outcomes for patients.

**Abstract:**

Uterine leiomyosarcoma (uLMS) is a rare and aggressive gynaecological malignancy. Surgical removal and chemotherapy are commonly used to treat uLMS, but recurrence rates are high. Over the last few decades, clarification of the genomic landscape of uLMS has revealed a number of recurring mutations, including *TP53, RB1*, *ATRX*, *PTEN,* and *MED12*. Such genomic aberrations are difficult to target therapeutically or are actively targeted in other malignancies, and their potential as targets for the treatment of uLMS remains largely unexplored. Recent identification of deficiencies in homologous recombination in a minority of these tumours, however, has provided a rationale for investigation of PARP inhibitors in this sub-set. Here, we review these mutations and the evidence for therapeutic avenues that may be applied in uLMS. We also provide a comprehensive background on diagnosis and current therapeutic strategies as well as reviewing preclinical models of uLMS, which may be employed not only in testing emerging therapies but also in understanding this challenging and deadly disease.

## 1. Introduction

Uterine leiomyosarcoma (uLMS) is a rare gynaecological malignancy arising in the smooth muscle layer of the uterus. It accounts for less that 2% of uterine malignancy [1], affecting approximately 0.6–0.8/100,000 women each year [2,3], but the uterus is the most common primary site of leiomyosarcoma in women, and uLMS accounts for more than 10% of all soft tissue sarcomas [4], so that is usually well represented as a distinct subgroup in clinical trials of metastatic soft tissue sarcoma. The 5-year survival rate of uLMS is 42–76% [5,6], yet uLMS accounts for 70% of uterine sarcoma deaths [7]. This is due to initial diagnosis often being made when the disease is already advanced, and metastasis has occurred. Recurrence following optimal surgical de-bulking is also common [8]. Moreover, due to the rare nature of this disease, there are few large cohort trials testing emerging therapies, and thus, advances in therapeutic management of uLMS have been slow to evolve.

## 2. Disease Characteristics and Epidemiology

uLMS are generally large tumours, often presenting at greater than 5 cm in diameter [9]. uLMS is typically diagnosed in the 6th decade of life, with an average age of 53 reported at diagnosis [6,10,11]. Carriers of inherited *TP53* and *RB1* mutations carry an increased risk of uLMS [12,13], and occasional cases have been reported with tamoxifen use [14,15], but in the majority of cases, no risk factors have been identified. Age at diagnosis, tumour size, and disease stage all impact survival outcome, with increases in each of these prognostic categories leading to poorer outcomes for women with uLMS [6,10,11]. In addition, African-American patients have lower survival rates than their White counterparts irrespective of treatment modalities [6,10,16]. As a tumour of the uterus, hormone receptor expression is often recorded at diagnosis, with both oestrogen receptor (ER) and progesterone receptor (PR) levels expressed at similar rates (35−87% and 17−80%, respectively) [17,18,19,20,21,22]. PR expression has been associated with improved PFS (20.8 months in PR positive cases compared to 8.1 months in PR negative) irrespective of tumour grade, whilst an improvement in PFS has only been observed in ER positive cases when adjusting for tumour grade [19]. Histopathological assessment is the standard method of diagnosis, as currently there is no test or imaging technique that allows for preoperative diagnosis. uLMS is classified into three subtypes: spindle-cell type and the less common myxoid and epithelioid types [23]. Spindle-cell-type uLMS contains spindle cells with nuclear pleomorphism and show tumour type coagulative necrosis and >10 mitoses per 10 high-power fields [9]. Myxoid tumours contain an abundance of myxoid stroma [24], whilst epithelioid tumours are comprised of round or polygonal cells with eosinophilic cytoplasm [23]. As some of these features are not unique to uLMS, differential diagnosis includes benign smooth muscle tumours, such as symplastic leiomyomas, leiomyomas with intravenous extension, mitotically active leiomyomas, smooth muscle tumour of uncertain malignant potential (STUMPs), and other uterine sarcomas, such as high-grade endometrial stromal sarcoma (HGESS), undifferentiated uterine sarcoma, perivascular epithelioid cell tumour (PEComa), and inflammatory myofibroblastic tumour (IMT) [25]. Diagnosis requires assessment of morphology and specific immunohistochemical stains to support the morphological diagnosis. Both benign and malignant smooth muscle tumours express smooth muscle markers, such as smooth muscle actin (SMA), Desmin, and Caldesmon [7]. uLMS tend to have greater expression of Ki−67, mutation type p53 staining, and stronger and more diffuse p16 expression compared with benign smooth muscle tumours [26,27,28]. STUMPs demonstrate positivity for smooth muscle markers similar to leiomyosarcoma and leiomyomas. Immunohistochemistry markers, such as p16, p53, Ki-67, p21, BCL2, ER, and PR, have been used to enhance the diagnosis and prognosis in these tumours without success [29]. Generally, STUMPs should have one of the criteria (diffuse moderate for severe cytological atypia, coagulative tumour necrosis, and increase mitotic activity) used for the diagnosis of leiomyosarcoma, but the histological abnormalities seen in a STUMP fall short of a diagnosis of leiomyosarcoma. Low-grade endometrial stromal sarcomas (LGESS) may show some positivity for smooth muscle markers and will also show diffuse positivity for CD10, ER, and PR, making them very difficult to differentiate from uLMS [30]. HGESS, however, show positivity for Cyclin-D1 and BCOR and will lack expression of CD10, ER, and PR [31]. HGESS also harbour *YWHAE::NUTM2A/B* gene fusions and BCOR rearrangements, which are diagnostic and not associated with uLMS [32], whilst the gene fusion *JAZF1::SUZ12* is most common in LGESS [33]. Beta-catenin may also be used to distinguish ESS from uLMS [34]. PEComas are composed of epithelioid and/or spindle cells and express at least one smooth muscle marker but differ from uLMS in their expression of HMB45 or Melan-A [35]. Detection of TFE3 rearrangement or fusion is helpful for the diagnosis of PEComa as distinct from uLMS [36]. Like uLMS, IMTs frequently express the smooth muscle markers SMA, Desmin, and Caldesmon [37]. ALK expression is also highly sensitive and specific for diagnosis of IMT; however, the degree of positivity varies, with some tumours showing only focal positivity [38]. Undifferentiated uterine sarcoma is a malignant mesenchymal tumour lacking evidence of specific lines of differentiation and therefore is a diagnosis of exclusion [39].

## 3. Treatment

Surgical removal of uLMS via hysterectomy is typically recommended based on data showing surgical removal with negative surgical margins leads to an increase in survival [6,10]. Importantly, resection of uLMS using power morcellation is associated with reduced survival (FDA warning April 2014; [40]) and thus should be avoided. The addition of bilateral salpingo-oophorectomy (BSO) is considered only if the patient is over the age of 50 years, as BSO is not associated with increased survival in patients with early-stage disease [6,10]. There has been little evidence to support adjuvant therapy following surgical removal of primary disease confined to the uterus. In a recent meta-analysis of 545 patients across nine studies, there was no survival advantage of adjuvant chemotherapy or radiation for patients with early stage, completely resected uLMS compared to observation alone [41]. Another recent analysis performed on 1030 cases collected by the National Cancer Database again showed no increase in survival for patients who received adjuvant chemotherapy (33% of cases), radiation (7.7%), or a combination of the two (6.2%) compared to observation alone following surgical removal of their primary tumour (53.1% of patients, suggesting that selection of patients for adjuvant therapy had taken place) [11]. In the metastatic setting, surgery is still considered beneficial. In a cohort of 96 patients with metastatic uLMS at presentation, treated at Memorial Sloan-Kettering Cancer Centre, the median overall survival (OS) of patients who received surgery with no residual disease was 31.9 months [42]. This is compared to just 5 months in those patients with inoperable disease. 

For women with advanced or inoperable uLMS, the principles of management of other soft tissue sarcomas are followed. Chemotherapeutic agents with efficacy include doxorubicin, combination gemcitabine and docetaxel, or trabectedin [43] and to a lesser extent dacarbazine or eribulin [44]. Doxorubicin remains the reference first-line agent following the GeDDiS trial that showed similar efficacy and better tolerance than gemcitabine/docetaxel [45]. The combination of gemcitabine/docetaxel is favoured over single-agent gemcitabine following a study of 122 women who received either combination therapy or gemcitabine alone, with a doubling of progression-free survival (PFS) reported in the combination arm (6.0 vs 3.2 months in the combination arm and single-agent arm, respectively) [46]. Gemcitabine/docetaxel combinations have been trialled in fixed-dose single-arm studies as both a first-line and second-line therapy in advanced uLMS, with PFS reported as 4.4 months and 5.6 months, respectively [47,48]. Ifosfamide, whilst having an important role in the management of other soft tissue sarcomas, has a limited role in LMS due to modest single-agent activity combined with the logistics of administration and its toxicity profile [49,50]. Trabectidin has demonstrated modest efficacy in uLMS, with one trial of 134 unresectable, locally advanced uLMS cases that had received prior chemotherapy, reporting a higher PFS in the trabectedin arm (4.0 months vs 1.5 months for the dacarbazine arm) [51]. A trial of 20 chemotherapy-naïve women with uLMS reported a PFS of 5.8 months, with mean OS of 26.1 months, for trabectedin treatment alone [52]. Similar results were recently observed in a Spanish cohort of patients [53]. For trabectedin in combination with doxorubicin as a first-line therapy in advanced uLMS, 28 out of 47 women achieved partial response, with a further 13 achieving stable disease [54]. Due to the PFS advantage demonstrated in unselected soft tissue sarcomas in the PALETTE trial, pazopanib may also be provided as a second-line therapy although this was not targeted to a specific biomarker [55].

Hormonal therapies have also been explored in uLMS given the high frequency of ER/PR expression in tumours, albeit in small cohorts. In a study involving 16 patients with ER/PR-positive advanced uLMS (12 of which were chemotherapy naïve) treated with an aromatase inhibitor, the authors reported a mean PFS of 14 months [56]. In another small study of newly diagnosed uLMS patients, all four patients receiving letrozole were progression free at both 12 and 24 months, whilst in the observational arm the proportion of individuals progression free at 12 and 24 months had reduced to 80% and 40%, respectively [57]. It was not required that patients be chemotherapy naïve in this study, but the inclusion criteria stipulated a disease-free period of at least five years from any other cancer. In the phase II PARAGON trial of anastrozole in a cohort of 32 patients with ER-positive uLMS, the PFS was 2.8 months, with one patient achieving stable disease for five years [58]. 

Targeted therapy, in the form of poly (ADP-ribose) polymerase (PARP) inhibitors (PARPi), has shown synthetic lethality in ovarian cancers bearing *BRCA1/2* mutations (as will be discussed later). With respect to uLMS, Seligson and colleagues recently found that mutations in *BRCA1/2* were enriched within the uterine subtype in a cohort of 170 LMS patients [59]. They and others have subsequently shown that such patients respond very well to PARPi [59,60]. Specifically, Seligson et al. reported durable stable disease in three out of four patients with *BRCA2*-mutated uLMS treated with olaparib, lasting for 15 months or more, and one partial response [59]. Hensley and colleagues also reported radiographic regression in five patients with *BRCA2*-mutated uLMS treated with PARPi, with one patient achieving a complete response [60].

Immunotherapies, such as the PD-1 blocking antibodies pembrolizumab and nivolumab, have been explored in relatively small cohorts over the last decade, as they have become increasingly popular in other solid tumours. PD-L1 expression, widely used as a biomarker to indicate immunotherapy response, has been reported as being positive in up to 70% of uLMS cases [61]. Two case studies involving patients with metastatic uLMS reported dramatic reductions in tumour burden and symptomatic disease with immune checkpoint inhibitor therapy [62,63], yet clinical trials on small patient cohorts have as yet failed to show any response to anti PD-1 therapy in uLMS [64,65]. However, in a basket study of doublet immunotherapy involving nivolumab and ipilimumab for four cycles followed by maintenance nivolumab [66], two patients had complete response and a third showed a partial response out of the five uLMS patients enrolled. This observation requires further investigation. 

Fifty-six clinical trials are currently listed on the ClinicalTrial.org website testing various combinations of chemotherapy, hormonal therapy, immunotherapy, and targeted agents in uLMS. The few clinical trials involving targeted agents are listed in Table 1 although many of these do not appear to specifically study uLMS. Hence, in keeping with the fact that most targeted therapy trials including uLMS patients are relatively non-specific, meaningful advances in treatment are lacking, and the survival of women with advanced uLMS remains dismal. Whilst there does not appear to be a single driver of this lethal tumour type, there are a number of genomic aberrations frequently observed in uLMS that have been successfully targeted in other more common cancer types. These will be explored in this review. 

## 4. Genomic Landscape

As uLMS is such a rare tumour type, molecular analysis has been limited. With so few samples collected at different sites throughout the world, large-scale analyses have often required input from multiple cohorts as shown in Table 2. To date, 23 and 158 uLMS tumours have undergone whole-genome sequencing (WGS) and whole-exome sequencing (WES), respectively, and been reported in the literature. An additional 434 uLMS samples have received a variation of exon capture, SNP array, or targeted panel sequencing. Collectively, the data show that *TP53* is the most frequently mutated gene (frequency of 26–92%), followed by *RB1* (27–88%), *ATRX* (24–34%), *PTEN* (19–75%), and *MED12* (12–21%). Mutations in the homologous recombination (HR) pathway have also been reported in uLMS, with mutations in *BRCA2* showing the highest frequency (7–60%).

uLMS tumours typically exhibit a tumour mutational burden (TMB) that is <10 mut/Mb, indicating they may not be strong candidates for immunotherapy given the findings from the recent KEYNOTE-158 study [83]. As with other trials, which defined high TMB as ≥10 mut/Mb [84,85], an objective response rate to pembrolizumab was observed in 29% of patients with a high TMB compared to just 6% of patients in the non-TMB-high group [83]. However, TMB is not indicative of response across all cancer types [86], and “TMB high” cut-offs vary widely [87]. Moreover, as mentioned previously, some success with immunotherapy in uLMS has been observed [62,63,66].

Large structural re-arrangements are common in uLMS. Choi and associates reported 21 cases of uLMS with complex structural rearrangements, 16 of which harboured chromoplexy or chromothripsis [78]. In their cohort of 10 uLMS cases, Chudsama et al. reported four had chromothripsis, one of which was a case that had received no treatment prior to analysis and yet had three affected chromosomes [81]. Structural rearrangements were also detected by Machado-Lopez et al., which resulted in gene fusions in 61.8% of cases, with *ATRX* the most common fusion partner [82].

The largest study to date on sarcomas, conducted by The Cancer Genome Atlas (TCGA) Program, compared uLMS to other sarcomas, confirming that uLMS is its own distinct tumour type even from its closest relative, soft-tissue LMS (ST-LMS). Compared to ST-LMS, uLMS had different methylation and mRNA expression signatures, with higher DNA-damage response scores and hypomethylation of ESR1 target genes [79]. ESR1 encodes the ER and therefore may explain why some uLMS patients are responsive to anti-endocrine therapies. 

Below, we discuss the most frequently mutated genes in uLMS and the emerging therapies targeting these aberrations, which could be potential future treatment options for uLMS patients.

## 5. TP53

Given their high frequency in all cancers, it is unsurprising that *TP53* mutations are the most common genomic aberration observed in uLMS. As with other tumour types [88], mutations in *TP53* in uLMS are typically missense mutations occurring in the DNA-binding domain (DBD). In a study assessing the genomic landscape of the largest cohort of uLMS patients (*n* = 215), 51% of *TP53* mutations were missense and found predominantly in the DBD [75]. Of the 40 *TP53* mutations reported by Choi and associates in their assessment of the Yale and TCGA uLMS cohorts, 23 (57.5%) were missense, with all except six such mutations located in the DBD [78]. Many of these missense mutations result in changes at critical residues within the DBD reducing proper protein–DNA interactions. Much research focus has therefore been aimed at restoring the conformational changes to the mutant protein enabling activation of key downstream genes. An example of this is APR-246, a methylated analogue of proline-rich membrane anchor 1, or PRIMA-1, which is converted to methylene quinuclidinone (MQ) once in the cell [89]. MQ then binds cysteine residues 124 and 277, which reactivates the stable confirmation of mutant p53 and thus DNA-binding capability [90]. APR-246 has recently shown some promise in a phase Ib/II clinical trial, wherein patients with *TP53*-mutant myelodysplastic syndromes or acute myeloid leukaemia were treated with a combination of APR-246 (eprenetapopt) and azacytidine [91]. The overall response rate in the 55-patient cohort was 71%, with 44% of patients achieving complete response. Several other clinical trials of APR-246 are underway or have recently been completed: notably two trials combining APR-246 with carboplatin and pegylated doxorubicin in recurrent high grade serous ovarian cancer (HGSOC) (NCT02098343) or pegylated doxorubicin alone in platinum-resistant HGSOC (NCT03268382). Fransson and colleagues have previously demonstrated synergy between APR-246 and carboplatin, cisplatin, and doxorubicin in primary *TP53*-mutant HGSOC [92], and thus, the results of the aforementioned clinical trials are keenly awaited. 

Another exciting avenue of therapeutically targeting mutant p53 is through the inhibition of WEE-1. In *TP53*-mutant cells the G_1_/S cell-cycle checkpoint is typically bypassed due to the absence of functional p53 protein; thus, cells become more reliant on the G_2_/M checkpoint [93]. Transition through this checkpoint and into mitosis is triggered by active cyclin dependent kinase 1 (CDK1). WEE-1 is a tyrosine kinase that inactivates CDK1 via phosphorylation, thereby halting the cell cycle at the G_2_/M checkpoint [94]. Inhibition of WEE-1 therefore removes the halt on mitosis, and cells already exhibiting a significant amount of DNA damage due to mutation of p53 incur further replication stress, resulting in mitotic catastrophe and cell death. It is thus predicted that just as PARPi show synthetic lethality in tumours bearing a *BRCA1/2* mutation, WEE-1 inhibition will be synthetically lethal in p53-mutant cancers. This has certainly been demonstrated both preclinically and clinically. In 2011, Rajeshkumar and associates reported that 25/49 pancreatic xenograft tumours with mutant p53 showed a 50% reduction in initial tumour mass when the WEE-1 inhibitor (WEE-1i) MK-1775 was added to gemcitabine treatment compared to just 7/55 treated with gemcitabine alone [95]. Pancreatic xenograft tumours that were wild type for p53 did not respond to either gemcitabine alone or the combination with MK-1775. Similar findings were observed in pancreatic xenograft tumours treated with a combination of MK-1775 and irinotecan or capecitabine, where effective tumour reductions were only observed in the mutant p53 models [96]. Due to its ability to induce replication stress, MK-1775 has also been shown to increase the cytotoxic capabilities of standard therapeutics in preclinical cancer models of the breast [97,98], cervix [99], oesophagus [100], ovary [101], and even in models of sarcoma [102]. In a clinical trial involving 121 *TP53*-mutant platinum-sensitive ovarian cancer patients, the addition of adavosertib (MK-1775) to carboplatin and paclitaxel improved progression-free survival by 1.9 months compared to placebo plus the combination chemotherapy. A clinical trial combining adavosertib with carboplatin in *TP53*-mutated ovarian cancer showed that this WEE-1i had the ability to resensitise tumours that were previously platinum resistant [103]. There are currently 14 active and 13 completed clinical trials involving a WEE-1i on the NIH Clinical Trials website, indicating the significant interest in the potential of this molecule for treating p53-mutant cancers. It should be noted, however, that p53 status cannot always predict response to WEE-1 inhibition. In a panel of eight sarcoma cell lines, Kreahling and associates showed sensitivity to MK-1775 in the nanomolar range irrespective of p53 status [102]. They then went on to show significant synergy when MK-1775 was added to gemcitabine therapy in four sarcoma cell lines, U2O5 (osteosarcoma), MG63 (osteosarcoma), A673 (Ewing sarcoma), and HT-1080 (fibrosarcoma), despite two being p53 wild type (U2O5 and HT-1080) [104]. Similar findings have also been reported in ovarian cancer cell lines [105] and lung and colorectal cancer cell lines [106]. In a clinical trial of 35 patients receiving daily adavosertib, *TP53*-mutant tumours were observed in both the responder and non-responder groups, noting that some responders were wild type for p53 [107]. 

## 6. RB1

*RB1*, the gene that encodes retinoblastoma protein (Rb), is a tumour suppressor with critical roles in many cellular processes, the most well characterised being a cell cycle regulator. In the resting cell state, Rb is hypophosphorylated and binds to E2F factors, restricting their ability to activate the transcription of genes required for S-phase entry, effectively halting the cell cycle at G_1_ [108]. In response to mitotic cues, Cyclins C, D, and E and their associated CDKs phosphorylate Rb, releasing E2F and allowing progression through G_1_ into S phase [108]. Often the second most commonly affected gene after *TP53* in uLMS, *RB1* is prone to homozygous deletion [75,77,78]. Deletion or inactivating events are classically hard to target therapeutically; however, again, synthetic lethality may be the key. Aurora kinase inhibitors have recently been shown to have selective sensitivity in Rb-null cell lines compared to Rb wild-type cells [109,110]. Aurora kinase A is involved in centrosome formation and mitotic spindle assembly, whilst Aurora kinase B assists with coordinating sister-chromatid cohesion during metaphase [111]. As Rb has also been proposed to contribute to mitotic fidelity through its association with E2F and E2F-independent mechanisms [112], the loss of Rb and the Aurora kinases A and B is thought to be too catastrophic for the cell. Clinical trials of Aurora kinase A inhibitor MLN8237 (Alisertib) have shown promise in ovarian cancer [113] as well as breast cancer, lung cancer, head and neck squamous-cell carcinoma, and gastro-oesophageal adenocarcinoma [114]. Following encouraging preclinical evidence of sensitivity to MLN8237 in the vulval LMS cell line SK-LMS-1 both in vitro and in vivo [115], a phase II clinical trial of Alisertib as a monotherapy was conducted in recurrent uLMS patients [116]. Of the 23 patients enrolled there, just one patient was progression-free at six months, with no partial or complete responses recorded. The authors also reported significant toxicity. In another multi-centre phase I/II trial of Alisertib as a single agent, 10 LMS (non-uterine) patients were included [117]. PFS at 12 weeks was 44% with OS reported as 72 weeks for this cohort. The exact molecular profile of these LMS tumours from the aforementioned trials were not disclosed, so patient responses were not stratified according to Rb status. In addition, both trials tested Alisertib as a single-agent. Aurora kinase inhibitors in combination with other agents are yet to be explored clinically in uLMS. 

Targeting the proteins that are up-regulated in response to Rb loss may be another mechanism of effective therapeutic control. As discussed, Rb is responsible for binding to and restricting the function of E2Fs. A pan-E2F inhibitor, HLM006474, was developed in 2008 showing efficacy in melanoma and triple-negative breast cancer cell lines [118]. More recently, HLM006474 has been shown to be effective at reducing cell viability in small-cell and non-small-cell lung cancer and synergises with paclitaxel [119]. Additionally, HLM006474 has been shown to inhibit growth of melanoma cells lines and synergises with BRAF-inhibitors [120]. Interestingly, the inhibition of tumour cell growth in this last study was observed only in p53 wild-type cells. This may predict reduced effectiveness of HLM006474 in uLMS, as *TP53* and *RB1* are frequently co-mutated, and may suggest why no clinical trials for HLM006474 have been initiated. 

## 7. ATRX and Alternative Lengthening of Telomeres (ALT) 

Alpha-thalassemia/mental retardation syndrome X-linked (ATRX) is a chromatin-remodelling factor that exists in a complex with death domain-associated protein (DAXX) and is essential for incorporating Histone 3.3 (H3.3) into telomeres [121,122,123,124]. Loss of ATRX or DAXX results in telomere instability and alternative lengthening of telomeres (ALT) [123]. Whilst frequent loss of *ATRX* has been observed in uLMS (39–52%), mutations in *DAXX* have been observed but are very rare (0–2%) [80,125]. Many aggressive tumours, such as glioblastoma multiforme [126] and diffuse intrinsic pontine gliomas [127], as well as those of mesenchymal origin [128], such as uLMS, display features of ALT. Indeed, ALT is the predominant telomere maintenance mechanism found in uLMS, with up to 71% of cases being ALT positive (ALT+) [80,125]. 

In normal cells, telomere maintenance mechanisms are constrained to prevent the formation of cancer. These mechanisms are either disrupted during the process of tumorigenesis or hijacked by cancer cells to enable them to avoid replicative senescence and acquire immortality [129]. Telomere maintenance in most cancer cells occurs due to reactivation of telomerase [130]. However, in 10–15% of cancers, some having a particularly poor prognosis [131,132], telomerase is suppressed, and telomere length is maintained through the ALT mechanism [133]. Cells that possess ALT extend their telomeres by copying other telomeres or extrachromosomal TTAGGG DNA fragments derived from telomeres through HR [134]. ALT telomeres display elevated replicative stress and DNA damage most likely associated with telomeric chromatin changes occurring as a result of mutations in genes that encode H3.3, ATRX, or DAXX [126,135,136]. It has been proposed that when the function of ATRX is lost, DAXX can no longer direct H3.3 to telomeres, leading to the formation of G-quadruplex structures that can give rise to replication stress [137,138]. HR at telomeres then occurs through the MRE11-RAD50-NBS1 complex, leading to ALT [138,139].

Almost all *ATRX*-mutant tumours are ALT+, but *ATRX* is not mutated in ~50% of ALT+ tumours, indicating alternative mechanisms driving this phenotype [125]. During DNA recombination, protein kinase ATR is recruited to telomeres by replication protein A, a protein persistently associated with telomeres in ALT+ cells. Therefore, it is hypothesised that ALT+ cancers may display sensitivity to ATR inhibitors. Studies investigating the use of ATR inhibitors (ATRi) to treat ALT+ cancers have produced conflicting results [140,141], so this warrants further assessment but may be a potential avenue to explore for the treatment of uLMS perhaps in combination with other relevant DNA-repair inhibitor therapies. The specialised DNA recombination thought to occur in ALT+ cells may also render them sensitive to inhibitors of DNA synthesis, such as PCNA and BLM inhibitors [142,143]. Furthermore, Liang et al. carried out a CRISPR screen in *ATRX*-knockout cells and identified components of the cell-cycle checkpoint as being important for cell survival. In particular, inhibition of WEE-1, which as discussed earlier is an important regulator of the G_1_/S and G_2_/M cell-cycle checkpoints, was found to be synthetically lethal in ATRX-deficient cells [144,145]. Not only does the presence of *TP53* mutations but also the presence of *ATRX* mutations suggests a subset of patients may benefit from WEE-1i. These have not yet specifically been tested in uLMS although women with uLMS should be encouraged to take part in basket trials of WEE-1i, such as NCT04158336 or NCT0476886. 

ATRX is also involved in transcriptional regulation but when recruited to promyelocytic leukaemia nuclear bodies by DAXX, this regulation of gene expression is inhibited [146]. Therefore, ATRX and DAXX function in a wide range of cellular processes both independently as chromatin remodellers as well as in complexes with H3.3 or PML. For example, ATRX associates with H3K9me at repetitive regions (other than telomeres), and reduced H3K9me levels as a result of loss of functional ATRX can give rise to stalled replication forks and genomic instability [139]. This may lead to activation of PARP and ATM, suggesting that ATRX-mutant cells may be responsive to PARPi [147]. ATRX has also been found to regulate the expression of Polycomb responsive genes through interacting with EZH2, indicating that ATRX-mutant cells may be sensitive to EZH2 inhibition [139,148,149]. 

## 8. PTEN

Phosphatase and tensin homologue deleted on chromosome 10 (PTEN) is a tumour suppressor whose expression is often lost in tumours [150,151,152]. PTEN is lost or deleted in about 66% of all uterine cancers and in 19% of uLMS specifically [75,153]. As a lipid phosphatase PTEN dephosphorylates phosphatidylinositol-3,4,5-phosphate (PIP_3_), a critical second messenger in the phosphatidylinositol-3-kinase (PI3K)/AKT signalling pathway [154]. PI3K phosphorylates PIP_2_ to give rise to PIP_3_, which is required to recruit AKT to the plasma membrane where it can be phosphorylated and activated by two kinases, PDK1 and mTORC2 [155]. Therefore, PTEN inhibits the PI3K pathway through converting PIP_3_ back to PIP_2_, thus regulating cell growth and survival. PTEN can also function as a protein phosphatase, where it has been found to dephosphorylate various proteins involved in cell proliferation and migration, including Cyclin D1, focal adhesion kinase (FAK), and Shc [156,157,158]. 

Apart from its phosphatase functions, PTEN also has phosphatase-independent functions in the nucleus, where it can regulate chromosome stability (through interacting with various chromosome factors, such as Centromere-Specific Binding Protein and anaphase-promoting complex or cyclosome), DNA repair (through directly interacting with p53, as well as regulating the expression of RAD51), and apoptosis (reviewed in [159,160]). Therefore, PTEN inhibits tumour development through multiple mechanisms and exploiting PTEN loss is the aim of many therapeutic strategies.

PTEN function is not only lost through mutation or genomic deletion of the *PTEN* gene but also through epigenetic and transcriptional silencing, post-transcriptional and post-translational regulation, and protein–protein interactions [161]. Therefore, the proportion of uLMS harbouring loss-of-function of PTEN could potentially be higher than 19%. Nevertheless, as previously described for loss of p53 and Rb, there are many challenges in therapeutically targeting loss of a protein. The PI3K/AKT/mTOR pathway is one obvious target in PTEN-deficient tumours. Cell lines with *PTEN* genetic alterations have displayed sensitivity to PI3K inhibitors (AZD6482), AKT inhibitors (MK-2206), and mTORC1 inhibitors (Temsirolimus) [162]. On the other hand, these cells are generally resistant to inhibitors of upstream mediators of PI3K pathway activity, such as receptor tyrosine kinases [162]. Many PI3K/AKT/mTOR inhibitors have been investigated in clinical trials for the treatment of PTEN-deficient tumours with mixed results depending on the molecular background (reviewed in [163,164]). Overall, mTORC1 inhibitors appear to have the greatest efficacy in these trials [163]. For example, mTOR inhibitors have demonstrated some efficacy in clinical trials in STS patients (no uLMS included in these trials) [165,166]. Combination of PI3Ki and the new TORC1/TORC2 inhibitors will likely have greater efficacy given that elevated PI3K/AKT signalling is a known feature of uLMS (due to PTEN loss and up-regulation of other genes in the pathway, such as *PIK3CA*, *AKT1/2/3*, and *RICTOR*) [79].

The interaction between PTEN and FAK may also be exploited therapeutically. Preclinical studies have indicated that PTEN-deficient models of uterine cancer were sensitive to the FAK inhibitor GSK2256098 in vitro and in vivo [167]. The link between PTEN and FAK has been studied in multiple different tumour types, and although many PTEN-deficient tumours display FAK activation, this is not always the case [164]. Whether this correlation is present in uLMS and therefore FAK inhibitors should be considered and needs to be explored. Furthermore, combining FAK and PI3K inhibitors may have greater efficacy, and preclinical studies involving this combination are also worth considering [168]. FAK is also a substrate of Src kinase and along with other Src substrates, such as c-KIT, EGFR, and PDGFR, has been found to be (over)expressed in uLMS [169,170]. Interestingly, the Src inhibitor dasatanib has been found to synergise with gemcitabine and docetaxel independently in uLMS cell lines [171].

Loss of nuclear PTEN function can also be exploited therapeutically. The role of PTEN in the DNA repair process of HR, through regulating RAD51 expression, may render deficient tumours sensitive to PARPi [172]. PARPi have previously demonstrated efficacy in PTEN-deficient endometrial cancers and warrants investigation in uLMS [173,174]. As has been mentioned previously and will become more apparent later in this review, many common aberrations in uLMS may indicate PARPi sensitivity, and this class of drug warrants further investigation in uLMS. 

## 9. MED12

The *MED12* gene on Xq13.1 encodes Mediator complex subunit 12 (MED12). The Mediator complex is composed of 25–30 proteins and plays a role in both activating and repressing gene transcription through mediating interactions between RNA Polymerase II (Pol II) at gene promoters and transcription factors at specific enhancers [175,176,177]. MED12 is a subunit of the Kinase module of Mediator, which consists of four proteins (CDK8, Cyclin C, MED12, and MED13) and is required for the kinase activity of the module [178,179]. Up to 86% of uterine leiomyomas have a mutation in *MED12* [180]; however, only about 20% of uLMS harbour a mutation in this gene [75,80,181,182], indicating alternative origins for this rare tumour. Most mutations occur in exons 1 and 2, the region required for Cyclin C interaction; however, mutations can also occur in other regions of the protein where they may result in “gain-of-function” of MED12, driving genomic instability [183].

Due to its role in essential developmental and cell fate determination processes, it is not surprising that MED12 deregulation can lead to cancer. Various molecular pathways relevant to tumour development, such as TGF-β signalling [184], Wnt signalling [185], and the p53 network [186], are affected in MED12-mutant cells. Importantly, MED12 inhibition has been found to confer resistance to a number of anti-cancer drugs in the context of specific mutations that are normally targetable [187,188]. Specifically, MEK/ERK activation has been found to remain high following treatment with ALK or EGFR inhibitors when MED12 is lost [189]. A similar effect has been seen in response to BRAF and MEK inhibitors, implicating MED12 in RAS-MEK-ERK signalling [184]. Resistance to the chemotherapeutic agents cisplatin and 5-FU has also been observed [184]. Further investigation found that cytoplasmic MED12 is able to interact with intracellular TGF-βR2, preventing it from being expressed on the cell surface. This leads to increased TGF-β signalling in MED12-mutant cells, resulting in activation of MEK and ERK (even in the presence of specific inhibitors), induction of EMT, and resistance to chemotherapeutic agents [184]. Small-molecule drugs that inhibit TGF-β signalling have been found to overcome drug resistance in MED12-deficient cells [184], indicating TGF-β inhibitors may be viable treatment options for uLMS patients harbouring mutations in *MED12*. On the other hand, methylation of MED12 has been found to render breast cancer cells sensitive to chemotherapeutic agents, indicating that MED12 has cancer type-specific functions [189]. Whether MED12-mutation confers drug resistance in uLMS that can be overcome by combination treatment strategies involving TGF-β inhibitors requires further investigation. 

The action of MED12 in TGF-βR2 regulation is independent of its role in the Kinase module of Mediator. In the Kinase module, MED12 is able to stimulate kinase activity through its interaction with Cyclin C [190]. Recently, it was found that loss of Cyclin C conferred resistance to the ATRi ceralasertib in mouse embryonic cells [191]. Whether this finding will translate to tumour cells is yet to be investigated; however, it suggests that using ATRi to target ALT in uLMS, as proposed earlier, may not be effective in tumours that also harbour *MED12* mutations. This is because most of the mutations in *MED12* cluster in Exon 1 and 2 [190], the region required for Cyclin C binding [75,80,181,182], and so may result in a similar lack of response to ATRi. Finally, Mediator and the Bromodomain-containing protein 4 (BRD4) have been found to have similar epigenetic functions at super-enhancers in acute myeloid leukaemia (AML) cells [192,193]. Although it is unknown whether BRD4 functionally compensates for loss of MED12, inhibition of BRD4 may also be a treatment strategy for uLMS harbouring a MED12 mutation. 

It is intriguing that *MED12* mutations are more frequent in uterine leiomyoma (up to 86%) than in uLMS (up to 22%). Furthermore, as *MED12* is located on the X-chromosome, the mutation could lie on the inactive X chromosome and thus be irrelevant. Both of these factors suggest *MED12* mutations may not function as driver mutations in uLMS, and so, therapeutics that are effective in the context of MED12-deficiency need to be carefully investigated.

## 10. Homologous Recombination Deficiency 

HR and classic non-homologous end joining are the primary repair pathways of DNA double-stranded breaks (reviewed by [194]). Of the two, HR is less error-prone, as it requires the template of a sister chromatid upon which accurate repair can occur. HR deficiency (HRD) is therefore the loss of this high-fidelity system of repair and leads to increases in mutation events that lead to loss of heterozygosity (LOH) and widespread genomic instability. Many proteins are involved in the HR process, including BRCA1, BRCA2, RAD51, and PALB2. Early studies reported modest frequencies of mutations in the HR pathway in uLMS tumours, particularly in *BRCA2*, but interest in the contribution of HR mutations in this cancer type has recently come under the spotlight due to promising clinical data for drugs targeting these aberrations as mentioned earlier [59,60]. As described above, Seligson and colleagues found that mutations in *BRCA1/2* were enriched within the uterine subtype in a cohort of 170 LMS patients [59]. Rosenbaum et al. supported this work in 2020, reporting a frequency of mutations in *BRCA2* of 9% in uLMS (*n* = 121) compared to 2% in ST-LMS (*n* = 90) [76]. Responsiveness to PARPi has been demonstrated preclinically in uLMS, with Choi et al. showing a favourable response to olaparib in a patient-derived xenograft of HRD uLMS [78]. HRD tumours are also typically sensitive to platinum therapy. Whilst cisplatin was trialled as a therapy for uLMS in the 1980s and 1990s, the findings did not indicate any patient benefit [195,196]. However, these studies were performed on small numbers of women and did not screen uLMS for mutations in the HR pathway; therefore, the full utility of cisplatin in HRD uLMS patients has not yet been thoroughly explored. 

Current methods for screening for mutations in the HR pathway in solid tumours involve panel tests, such as the QIAseq Targeted DNA BRCA1 and BRCA2 Panel and FoundationOne Panel, with more challenging options such as WES or WGS typically reserved for more complex or advanced cases in the research setting. Given that *BRCA1/2* mutations (predominantly *BRCA2*) only account for ~10% (but can be as frequent as 60% [81]) of uLMS cases, panel tests appear to be a reasonable alternative. However, hallmarks of genomic instability, such as LOH and telomeric allelic imbalance, are more reliably detected through WGS. The Myriad myChoice CDx test aims to cover these two aspects by calculating a genomic instability score based on the aforementioned measures of genomic instability as well as large-scale state transitions *and* mutations in *BRCA1* and *BRCA2*; however, at USD 4000, this test may not be much less expensive than WGS but may be more easily available. 

## 11. Other Genomic Characteristics

Epigenomic data on 27 uLMS cases published by TCGA indicated that a unique methylation pattern was observed despite hypomethylation of ESR1 target genes. Overall, uLMS appeared to be hypermethylated compared to ST-LMS [79]. This suggests uLMS may be sensitive to epigenetic modulators, such as DNA Methyltransferase inhibitors (DNMTi). In a preclinical study, the uLMS cell line SK-UT-1 was found to be sensitive to the DNMTi guadecitabine in vitro and in vivo. In vitro efficacy appeared to be correlated with methylation status, as the comparatively hypomethylated cell line SK-LMS-1, which interestingly is a non-uterine LMS cell line, was less responsive [197]. *SHARPIN*, a protein coding gene involved in TNF signalling, was recently shown to be amplified in uLMS via WES [198]. Investigations of *SHARPIN* mutations in the TCGA dataset revealed amplification of *SHARPIN* leads to decreased overall survival. Knockdown of *SHARPIN* in uLMS cell lines lead to decreased cell proliferation and colony-forming ability [198].

## 12. Preclinical Models of uLMS

**Figure 1:**
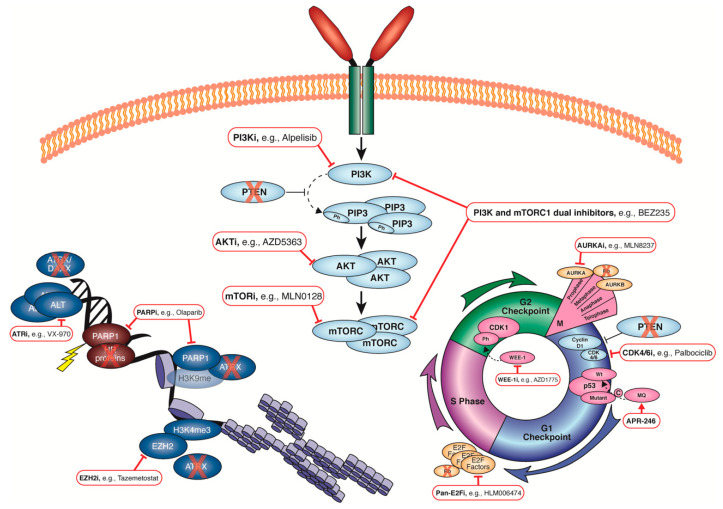
Summary of the common genetic aberrations in uLMS and their potential therapeutic targets.

In this review, we detail the frequent genomic aberrations observed in uLMS and the possible targeted agents that are worth investigating further in the context of uLMS (Figure 1, Table 3). In line with this, the field requires well-characterised and validated models of uLMS that represent the heterogeneity observed in patients. The immortalised cell lines SK-UT-1 and SK-UT-1B have been well annotated [81] and are utilized often in uLMS research. In 2008, Press and colleagues reported the generation of a uLMS PDX that reached three generations [199]. Ten uLMS patient-derived xenograft (PDX) models were successfully generated by Cuppens and associates more recently [200], five of which were used in a subsequent study to show the benefit of inhibiting mTOR and PI3K signalling in uLMS (4/5 PDX models showed reduction/stabilisation of tumour growth) [201]. Two uLMS PDX models reported recently with mutations in the HR pathway were treated with single-agent olaparib, pan-PI3K inhibitor, or a BET bromodomain inhibitor, with all three therapeutics reducing tumour growth [78]. The HR status of the other PDX models reported by Cuppens or Press was not included. 

Genetically-engineered mouse models (GEMMs) of uLMS have also been attempted. Conditional inactivation of p53 in the mouse reproductive tract through expression of Cre recombinase under the control of the anti-Mullerian hormone type II receptor (*Amhr2*) gave rise to uLMS in about 50% of mice, with frequency increasing to 82% when BRCA1 was also inactivated [203]. Transgenic mice expressing a growth factor called Cripto-1 (CR-1) under control of the *MMTV* promoter to activate the Wnt pathway also activated the Src/AKT pathway and gave rise to uLMS in 20% of mice [204]. uLMS GEMMs have also been generated by crossing mice with the T antigens of the SV40 region (*SVER*) transgene (which encodes the three T antigens of SV40–SV Large T (SVLT), small t, and 17kT) targeted to the mouse *Beta-actin* locus with mice containing a *Cre* transgene downstream of the *Hsp70-1* heat-shock promoter [205]. Despite almost ubiquitous expression of SVLT, tumour development was restricted to the uterus (with tumours showing uLMS morphology) in female pups and seminal vesicles in male pups. The potential of these GEMMs to drive forward our understanding of uLMS is clear; however, recent advances in this space are lacking, and treatment studies have to date not been reported. 

## 13. Conclusions and Future Perspectives 

The high rate of genetic aberrations in uLMS and the increasing advances in targeted therapies provide many possibilities for the future treatment of uLMS. PARPi are one of the most significant advances in the uLMS space, with striking favourable responses akin to what is observed in other HRD gynaecological malignancies. In addition, despite HRD only accounting for a small proportion of cases, PARPi may also be beneficial in HR proficient but PTEN and/or ATRX deficient tumours as discussed above, thus potentially providing benefit to a greater subset of patients. Similarly, PI3K, mTORC1/2, and WEE-1 inhibitors are likely to impact a larger subset of women with uLMS, as they target a number of specific aberrations commonly observed in uLMS and may also provide an opportunity for advantageous combinatorial therapy with PARPi. 

As observed for other cancer types, it is unlikely that one therapeutic approach will be of use for all women with uLMS. The large variability in mutation frequencies indicates that a personalised approach may be more beneficial. Whilst this review explores the different mutations and the therapies that can target them in isolation, we have not yet accounted for the complex interactions between concurrent mutations or the ripple effects in downstream signalling pathways that arise as a result of small molecule inhibition. It should also be noted that the molecular profiling of uLMS is still in its infancy compared with other tumour types. With less than 200 reported cases of WGS and WES, researchers have not yet been able to perform the deep sequencing on large data sets that is likely required to understand this clinically elusive, rare tumour type and the molecular subsets within uLMS. In light of this, clinicians should consider WGS or WES in their diagnostic approach to management of women with uLMS not only to discover the potentially actionable mutations described in this review but also to contribute to the global effort in uncovering the multifaceted biology that underpins uLMS.

## Figures and Tables

**Table 1:** List of clinical trials including uLMS patients.

Identifier	Target	Interventions	Notes, References
NCT01637961	AURKA	Alisertib	Phase II; uLMS; completed, no response
NCT00378911	VEGFR1/2/3, PDGFRa/b, c-KIT, RET, GCSFR, FLT-3	Sunitinib malate	Phase II; uLMS; completed, but no results reported
NCT02428192	CTLA4, PD-1	Ipilimumab and Nivolumab	Phase II; uLMS; active, no response [67]
NCT03880019	PARP	Olaparib and Temozolomide	Phase II; uLMS; active, no results [67]
NCT01012297	VEGF	Bevacizumab	Phase III; no response [68]
NCT02203760	VEGFR1/2/3, PDGFRa/b, c-KIT	Pazopanib + Gemcitabine	Phase II; metastatic uLMS or UCS; recruiting
NCT02601209	VEGFR1/2/3, PDGFRa/b, c-KIT, mTORC1/mTORC2	Pazopanib or Sapanisertib	Phase I/II; unresectable LMS and STS; slight benefit with pazopanib [69]
NCT04200443	VEGFR2, c-Met, AXL, RET	Cabozantinib	Phase II; unresectable LMS and STS; recruiting
NCT00659360	Src, Lck, Fyn, Lyn, c-Yes, Blk, Abl EGFRmut	Saracatinib	Phase II; LMS, STS, and uterine sarcoma (US); no response
NCT00245102	VEGFR1/2/3, PDGFRb, RAF-1, BRAF (wt and mut), c-KIT, FLT-3	Sorafenib tosylate	Phase II; LMS, STS, and US; no response
NCT00390234	VEGF-Trap	Ziv-Aflibercept	Phase II; uLMS, UCS, and US; no response
NCT01442662	VEGFR1/2/3, PDGFRa/b, c-KIT	Pazopanib + Gemcitabine	Phase II; second-line LMS; no response [70]
NCT00474994	VEGFR1/2/3, PDGFRa/b, c-KIT, FLT-3, CSF1R, RET	Sunitinib malate	Phase II; metastatic/recurrent sarcomas; SD best response [71]
NCT00526149	PLK1/2/3, BRD4	BI-2536	Phase II; metastatic/recurrent solid tumours; no response [72]
NCT00006357	PDGFR, c-KIT, Abl	Imatinib mesylate	Phase I/II; recurrent/refractor STS; no response [73]
NCT00053794	AKT	Perifosine	Phase II; metastatic STS; no response [74]

**Table 2:** Summary of published uLMS patient cohorts for genomic/genetic analysis.

Cohort	*n*	Sequencing (How Many, What Type, Who Analysed/Reported)
MSKCC	128	*n* = 80 exon capture [60,75] *n* = 121 exon capture [76]
Cuppens	62	*n* = 2 WGS [77] *n* = 62 SNP array [77]
Yale	55	*n* = 11 WGS [78] *n* = 55 WES, RNA-Seq [78]
MSK/Genie	51	*n* = 51 exon-targeted seq [75]
Dana-Faber	39	*n* = 39 exon-targeted seq [75]
OSU	34	*n* = 35 FoundationOne panel test [59]
TCGA	31	*n* = 10 WGS [78,79] *n* = 27 WES, RNA-Seq [59,78,79] *n* = 31 exon-targeted seq [75]
Helsinki	19	*n* = 19 WES [80]
Chudasama	10	*n* = 10 WES [81]
U Mich	8	*n* = 8 exon-targeted seq [75]
Vanderbilt	7	*n* = 7 exon-targeted seq [75]
Spain	44	*n* = 44 WES, RNA-Seq [82]

WGS, whole-genome sequencing; SNP, single nucleotide polymorphism; WES, whole-exome sequencing.

**Table 3:** Summary of the potential therapeutic targets that arise due to genetic aberrations frequently occurring in uLMS.

Aberration	Frequency	Therapeutic Target	Potential Drugs
*TP53*	26–92%	Mutant p53 [90]	APR-246 [91]
		WEE-1 [95]	AZD1775/MK-1775 [103,107]
*RB1*	27–88%	AURKA [109,110]	MLN8237/Alisertib [113,114,117]
		E2F [108]	HLM006474 [118,119]
*ATRX*	24–34%	ATR	VE-821 [141], VX-970, AZD6738
		EZH2 [139,148,149] BLM PCNA PARP WEE-1	Tazemetostat, GSK-126 ML216 [142] T2AA [143] PJ34 [147] AZD1775 [144,145]
*PTEN*	19–75%	PI3K [163,164]	AZD6482 [162], Buparlisib
		AKT [163,164]	MK-2206 [162], AZD5363
		mTORC1 [163,164] SRC FAK	Temsirolimus [162,166], Everolimus, Ridaforolimus [165] Dasatinib [172] VS-4718, VS-6063, PF-573228, PF-562271, GSK2256098 [168]
		PARP	KU0058948 [174], Olaparib [175]
*MED12*	12–21%	TGF-βR	LY2157299 [185]
		BET [193]	JQI
*HRD*	7–60%	PARP [78,81,202]	Olaparib [59,60]

## Data Availability

Not applicable.
